# Hepatitis A Virus Outbreaks Associated with Drug Use and Homelessness — California, Kentucky, Michigan, and Utah, 2017

**DOI:** 10.15585/mmwr.mm6743a3

**Published:** 2018-11-02

**Authors:** Monique Foster, Sumathi Ramachandran, Katie Myatt, Danielle Donovan, Susan Bohm, Jay Fiedler, Bree Barbeau, Jim Collins, Douglas Thoroughman, Eric McDonald, Jonathan Ballard, Jeffrey Eason, Cynthia Jorgensen

**Affiliations:** ^1^Division of Viral Hepatitis, National Center for HIV, Viral Hepatitis, STD, and TB Prevention, CDC; ^2^Kentucky Department for Public Health; ^3^Michigan Department of Health and Human Services; ^4^Utah Department of Health; ^5^San Diego County Health and Human Services Agency, San Diego, California; ^6^CEFO Program, Division of State and Local Readiness, Center for Preparedness and Response, CDC.

During 2017, CDC received 1,521 reports of acute hepatitis A virus (HAV) infections from California, Kentucky, Michigan, and Utah; the majority of infections were among persons reporting injection or noninjection drug use or homelessness. Investigations conducted by local and state health departments indicated that direct person-to-person transmission of HAV infections was occurring, differing from other recent, large HAV outbreaks attributed to consumption of contaminated commercial food products. Outbreaks with direct HAV transmission among persons reporting drug use or homelessness signals a shift in HAV infection epidemiology in the United States, and vaccination of these populations at high risk can prevent future outbreaks.

## Epidemiologic Investigation

Outbreak cases were defined as those meeting the 2012 CDC-Council of State and Territorial Epidemiologists’ (CSTE) definition of acute hepatitis A infection,* having a specimen matching an outbreak strain, or an epidemiologic link to a previously identified case. Local and state health department personnel reviewed clinical charts and interviewed patients using standard questionnaires that evaluated risk factors associated with infection, including recent drug use, sexual history, housing status, recent international travel, and contact with another person with HAV infection.

Among states reporting increases in HAV infections to CDC outside or inside the National Notifiable Disease Surveillance System, only California, Kentucky, Michigan, and Utah reported sustained within-state transmission. This report includes outbreaks that occurred during 2017 in these four states. Additional cases reported from other states were excluded because they were attributed to HAV exposure during travel to one of the four outbreak states, and because prolonged, ongoing transmission did not occur in the other states.

During 2017, a total of 1,521 outbreak-associated HAV cases were reported from California, Kentucky, Michigan, and Utah, with 1,073 (71%) hospitalizations and 41 (3%) deaths (Table 1). Among patients for whom clinical or laboratory records were available for review, 42 (3%) had confirmed or probable hepatitis B virus coinfection, and 341 (22%) had confirmed or probable hepatitis C virus coinfection. Overall, 866 (57%) patients reported drug use, homelessness, or both (Table 2). Among all cases, 818 (54%) had an indication for hepatitis A vaccination before becoming infected (i.e., using drugs or being men who had sex with men [MSM]) as recommended by the Advisory Committee on Immunization Practices (ACIP) (*1*).

**TABLE 1:** Demographic and clinical characteristics of hepatitis A outbreak–associated cases, by state — four states, 2017

Characteristic	California	Kentucky	Michigan	Utah	Total
**Total cases, no.**	**682**	**59**	**632**	**148**	**1,521**
Male, no. (%)	471 (69)	39 (66)	412 (65)	97 (66)	**1,019 (67)**
Median age, yrs (range)	42 (5–87)	36 (1–84)	41 (<1–90)	38 (22–83)	**—**
Earliest onset, date	01/17/2017	08/29/2017	01/05/2017	05/08/2017	**—**
**Outcome**					
Hospitalized, no. (%)	442 (65)	45 (76)	508 (80)	78 (53)	**1,073 (70)**
Died, no. (%)	21 (3)	0	20 (3)	0	**41 (3)**
**Comorbidities**					
Hepatitis B infection, no. (%)	10 (1)	4 (7)	16 (3)	12 (8)	**42 (3)**
Hepatitis C infection, no. (%)	116 (17)	29 (49)	165 (26)	31 (21)	**341 (22)**

**TABLE 2:** Risk exposures of hepatitis A outbreak–associated patients, by state — four states, 2017

Reported risk exposure	No. (%*)				
	California	Kentucky	Michigan	Utah	Total
Homelessness and drug use	247 (36)	27 (46)	64 (10)	75(51)	**395 (26)**
Homelessness only	65 (10)	3 (5)	7 (1)	5 (3)	**78 (5)**
Homelessness, drug use unknown	43 (6)	2 (3)	2 (0.3)	5 (3)	**51 (3)**
Drug use only	67 (10)	11 (19)	165 (26)	28 (19)	**265 (17)**
Drug use, homelessness unknown	11 (2)	1 (2)	58 (9)	7 (5)	**77 (5)**
Neither homelessness nor drug use	190 (28)	13 (22)	286 (45)	15 (10)	**504 (33)**
Men who have sex with men	18 (3)	4 (7)	61 (10)	1 (0.7)	**81 (5)**
Unknown	59 (9)	2 (3)	27 (4)	13 (9)	**114 (8)**

* Percentage totals sum >100 because of men who had sex with men being included independently and as part of “homelessness,” “drug use,” and “neither homeless nor drug use” categories.

## Laboratory Investigation

When available, serum specimens from patients who met the CSTE case definition were sent to CDC’s Division of Viral Hepatitis laboratory for HAV RNA isolation, genotyping, and genetic characterization. HAV RNA was extracted from immunoglobulin M antibody-positive serum samples and used to amplify and Sanger-sequence a 315–base-pair fragment of the VP1/P2B region (*2*). During 2017, 1,169 specimens from outbreak-associated cases from the four affected states were sent to CDC for additional testing. A total of 1,054 (90%) specimens had HAV confirmed by polymerase chain reaction, 1,014 (96%) of which tested positive for a genotype 1b viral strain. The strains circulating in California, Kentucky, and Utah were genetically different from those circulating in Michigan.

## Public Health Response

CDC worked with affected local and state health departments to apply control measures through health advisories, public education, and vaccination clinics that provided outreach and vaccination to the targeted populations. Vaccine was administered in jails, emergency departments, syringe exchange programs, drug treatment facilities, and homeless shelters. In certain jurisdictions, investigation teams also visited homeless encampments to educate and vaccinate unsheltered homeless groups. Although reporting of new outbreak cases in California has ended, new case investigations continue in Kentucky, Michigan, and Utah. Vaccination campaigns also continue for MSM and persons who use drugs or report homelessness in the affected states.

## Discussion

After the introduction of hepatitis A vaccine in 1996, the incidence of reported HAV infection steadily decreased in the United States until 2011 and then stabilized at an annual average of approximately 1,600 reported cases, mostly among international travelers returning from countries with endemic HAV or as part of foodborne outbreaks (*1*,*3*). HAV outbreaks among illicit drug users were common in the prevaccine era; during the mid-1980s, drug users accounted for >20% of all HAV cases reported to CDC (*3*,*4*). However, large community outbreaks within this population rarely occurred after 1996, when hepatitis A vaccine was first recommended for persons who use illicit drugs (*3*,*4*).

Person-to-person transmission of HAV between those who report drug use or homelessness can result from unsafe sanitary conditions or specific sexual contact or practices, or it can be parenterally transmitted through contaminated needles or other injection paraphernalia (*4*–*6*). Transient housing, economic instability, limited access to health care, and distrust of government services make outbreaks among affected populations more difficult to control, requiring tailored comprehensive public health interventions that address their specific circumstances and needs (*5*–*7*).

During 2016, U.S. hospitalization and mortality rates associated with HAV infections were 42% and 0.7%, respectively (*3*). Increased hospitalization and mortality rates observed in the 2017 HAV outbreaks might be attributable to preexisting illnesses, including chronic hepatitis B and hepatitis C infections, other comorbidities, age, and risk behaviors common among persons reporting drug use and homelessness (e.g., heavy alcohol use) (*8*).

Increasingly, investigations of HAV infections are using molecular epidemiology to confirm outbreaks (*2*). Laboratory data, when combined with reliable epidemiologic data, can be effective in understanding transmission networks, particularly among populations distrustful of investigators. The majority of surveillance specimens tested by CDC’s laboratory before 2017 were genotype 1a, the most common genotype in North and South America, but expansion of genotype 1b attributed to the current outbreaks is leading to increased detection of this previously uncommon genotype (*2*,*9*).

Vaccination rates among existing ACIP-identified risk groups are unknown but are believed to be low (*10*). On October 24, 2018, ACIP voted unanimously to add “homelessness” as an indication for ACIP-recommended HAV vaccination (*1*).^†^ Although the outbreak has ended in California, hepatitis A outbreaks among persons reporting drug use or homelessness continue in Kentucky, Michigan, and Utah, and, as of October 12, 2018, >7,000 outbreak associated cases have been reported from 12 states.^§^

Increasing vaccination coverage among all at-risk groups recommended by ACIP to receive hepatitis A vaccine might halt ongoing outbreaks and prevent future large community outbreaks (*1*).

CDC has recommended that local health jurisdictions experiencing HAV outbreaks among persons who report drug use or homelessness ensure procedures are in place for identifying these risk factors and that these groups are vaccinated against HAV infection.^¶^ State and local health departments and CDC should be notified of any new suspected clusters of acute HAV infections.

**Summary**

**What is already known about this topic?**

Hepatitis A is a vaccine-preventable viral infection of the liver that is commonly transmitted through consumption of microscopic amounts of feces. Outbreaks of hepatitis A infections are infrequent in the United States and are typically associated with contaminated food items.

**What is added by this report?**

During 2017, California, Kentucky, Michigan, and Utah reported 1,521 hepatitis A infections, mostly among persons who reported drug use or homelessness, signaling a shift in hepatitis A epidemiology from point-source outbreaks associated with contaminated food to large community outbreaks with person-to-person transmission.

**What are the implications for public health practice?**

Increasing vaccination among groups at risk for hepatitis A infection might halt ongoing outbreaks and prevent future outbreaks.

## Footnotes

* https://wwwn.cdc.gov/nndss/conditions/hepatitis-a-acute/case-definition/2012.

† https://www.cdc.gov/vaccines/acip/meetings/downloads/agenda-archive/agenda-2018-10-508.pdf.

§ https://www.cdc.gov/hepatitis/outbreaks/2017March-HepatitisA.htm.

¶ https://emergency.cdc.gov/han/han00412.asp.
